# Intact lung tissue and bronchoalveolar lavage fluid are both suitable for the evaluation of murine lung microbiome in acute lung injury

**DOI:** 10.1186/s40168-024-01772-6

**Published:** 2024-03-18

**Authors:** Lijun Zheng, Chengjun Liu, Hongjing Wang, Jun Zhang, Lejiao Mao, Xiaomei Dong, Siyao Hu, Na Li, Dandan Pi, Jingfu Qiu, Feng Xu, Chengzhi Chen, Zhen Zou

**Affiliations:** 1 Molecular Biology Laboratory of Respiratory Disease, Institute of Life Sciences, Chongqing Medical University, Chongqing, 400016 People’s Republic of China; 2 Department of Pediatric Intensive Care Unit, Children’s Hospital of Chongqing Medical University, Chongqing, 400014 People’s Republic of China; 3 Department of Health Laboratory Technology, School of Public Health, Chongqing Medical University, Chongqing, 400016 People’s Republic of China; 4 Department of Occupational and Environmental Health, School of Public Health, Chongqing Medical University, Chongqing, 400016 People’s Republic of China; 5 Research Center for Environment and Human Health, School of Public Health, Chongqing, 400016 People’s Republic of China

**Keywords:** Lung microbiome, Acute lung injury, 16S rRNA, Actinobacteriota

## Abstract

**Background:**

Accumulating clinical evidence suggests that lung microbiome is closely linked to the progression of pulmonary diseases; however, it is still controversial which specimen type is preferred for the evaluation of lung microbiome.

**Methods and results:**

To address this issue, we established a classical acute lung injury (ALI) mice model by intratracheal instillation of lipopolysaccharides (LPS). We found that the bacterial DNA obtained from the bronchoalveolar lavage fluid (BALF), intact lung tissue [Lung(i)], lung tissue after perfused [Lung(p)], and feces of one mouse were enough for 16S rRNA sequencing, except the BALF of mice treated with phosphate buffer saline (PBS), which might be due to the biomass of lung microbiome in the BALF were upregulated in the mice treated with LPS. Although the alpha diversity among the three specimens from lungs had minimal differences, Lung(p) had higher sample-to-sample variation compared with BALF and Lung(i). Consistently, PCoA analysis at phylum level indicated that BALF was similar to Lung(i), but not Lung(p), in the lungs of mice treated with LPS, suggesting that BALF and Lung(i) were suitable for the evaluation of lung microbiome in ALI. Importantly, Actinobacteria and Firmicutes were identified as the mostly changed phyla in the lungs and might be important factors involved in the gut-lung axis in ALI mice. Moreover, Actinobacteria and Proteobacteria might play indicative roles in the severity of lung injury.

**Conclusion:**

This study shows both Lung(i) and BALF are suitable for the evaluation of murine lung microbiome in ALI, and several bacterial phyla, such as Actinobacteria, may serve as potential biomarkers for the severity of ALI.

Video Abstract

**Supplementary Information:**

The online version contains supplementary material available at 10.1186/s40168-024-01772-6.

## Introduction

The lung is previously considered to be a sterile environment and is initially excluded from the Human Microbiome Project priority organ system list [1–4]. However, with the advent of next-generation sequencing technology, the concept of lung asepsis has been overturned, and microbial communities do exist in the lung [3, 5–8]. Extensive studies have revealed the role of gut microbiome contributing for disease process, but the research on lung microbiome is scare [1, 6, 9]. Among the studies on lung microbiome published from 2015 to 2018, only 20% were animal studies, and most of them were performed on patients. Moreover, there is even no uniform specimens for studies of the lung microbiome [9].

Sputum, BALF, and lung tissue are potential specimens for the study of lung microbiome. Due to sputum specimens which are easily affected by upper respiratory tract flora, therefore BALF, lung tissue is more suitable for the evaluation of lung microbiome. Although both BALF and lung tissues can be obtained from animal models, lung tissues are almost unachievable from clinical patients in most cases. Recent studies using BALF have shown that lung microbiome is changed in respiratory diseases [10, 11], such as asthma, idiopathic pulmonary fibrosis (IPF), and chronic obstructive pulmonary disease (COPD), suggesting that pulmonary microbiome may be associated with lung diseases progression and largely extending the knowledge of lung microbiome [9, 12–22].

The lung microecology has the characteristics of heterogeneity and low biomass, which is highly variable and may cluster by environment [23]. Previous study indicated that whole lung tissue was the preferred specimen type for murine lung microbiome studies, because bacteria detected in BALF had similarity with those of procedural, reagent, and sequencing controls [24]. Strangely, why BALF specimens are suitable for the determination of lung microbiome in patients but not in murine model? If we enlarge the number of mice, can we conquer the issues that the highly variable of microbiome in BALF specimens? If we compare the lung microbiome using BALF from clinical patients and animal model, do we get more accurate information? In the current study, we also want to determine whether the lung tissues after perfusion are suitable for lung microbiome study. In addition, we collected the feces specimens to observe the potential link between gut microbiome and lung microbiome.

A classical acute lung injury (ALI) mouse model was established by intratracheal instillation of lipopolysaccharide (LPS) according to our previous study [25], and 16S rRNA gene sequencing is applied to determine the composition of microbiome. Consistent with previous studies, our study demonstrated that intact lung tissues [Lung(i)], but not perfused lung tissues [Lung(p)], are suitable for lung microbiome determination. Interestingly, our study found that BALF specimens from one mouse treated with LPS are suitable for the evaluation of lung microbiome; however, the number of mice should be larger than four in control group. More importantly, we identified that several phyla (such as Actinobacteriota) had most significant changes upon lung injury occurred and might be associated with the severity of lung injury. This study provides novel insight that BALF specimens are suitable for the evaluation of lung microbiome, which would contribute to bridge the gap between clinical research and animal research in the role of lung microbiome in ALI.

## Methods

### Mice

This study has been approved by Chongqing Medical University’s Animal Care Ethics Committee (IACUC-CQMU-2023–0088). In general, 7-week-old male C57BL/6 mice (19–21 g) were purchased from Slack Jingda Laboratory Animal Co., Ltd. (Slack Jingda Laboratory Animal Co., Ltd., Hunan, China) and kept at the Animal Laboratory Center of Chongqing Medical University. All the mice were housed in individual vented cages with free access to food and water. In order to avoid the influence of the feeding environment on the experiment, mice were randomly assigned to each experimental group. The LPS instillation procedure was performed as previously described [25]. Briefly, mice were anesthetized with avertin (2,2,2-tribromoethanol, Sigma-Aldrich, T48402) by intraperitoneal injection at 360 mg/kg. After anesthetization, mice were placed on a foam board tilted at 45°, and the limbs and head were immobilized. After the exposure of the trachea, LPS (0.5 mg/kg, in 50-μl PBS) or PBS were injected by a needle and then followed by 150-μL air injection to ensure the uniform diffusion of LPS or PBS in the lung tissue. Mice with regular breath were then transferred to a thermostatic pad until they recovered from anesthesia. All the mice were sacrificed after 3 days.

### Specimens’ selection and processing

In order to avoid the contamination of the specimens, the whole collection process was completed in the ultraclean worktable. Mouse feces were collected in advance of the collection of lung specimens. The mice were randomly divided into lung tissue (intact), BALF, or lung tissue (perfused) groups. For lung tissue (intact) group, the lung tissues were collected directly; for lung tissue (perfused) group, the lung tissues were collected after the BALF collection; and for BALF group, the lungs were perfused three times through the trachea with 1-mL PBS to obtain BALF. The BALF was then centrifuged at 400 × g for 5 min at 4 °C, and the supernatants were carefully aspirated to another centrifugation at 15,000 × g for 15 min at 4 °C. The precipitates were collected for the detection of lung microbiome [1, 26, 27]. Between the collection of each tissue, the operating apparatus was sterilized. All specimens were frozen in liquid nitrogen and stored at − 80 °C until DNA isolation.

### Cell count and protein concentration in BALF specimen

The BALF was centrifugated at 400 × g for 5 min at 4 °C, the pellets were collected and resuspended with red blood cell lysate and then followed with another centrifugation at 2000 rpm at 4 °C for 10 min. The supernatant was used to determine the protein concentrations by a BCA assay kit (Beyotime, P0009), according to the manufacturer’s procedure; the pellets were resuspended with 1-mL PBS, and the cell count in BALF was determined by automated cell counter (Countess II, Invitrogen).

### Hematoxylin and eosin staining

Briefly, the lung tissues were immersed in 10% paraformaldehyde and then embedded in paraffin. The H&E staining was performed by a commercial kit (Solarbio, G1120), according to the manufacturer’s instruction. The H&E images were captured under microscope (Olympus, DP74).

### DNA extraction and PCR amplification

Total specimens’ microbial genomic DNA was extracted using the E.Z.N.A.® Soil DNA Kit (Omega Bio-tek, Norcross, GA, USA) according to the manufacturer’s instructions. To detect contamination during the procedure, empty EP tubes, PBS for lung tissue lavage, and buffer for extraction and amplification were included together as controls (*n* = 3). The quality and concentration of DNA were determined by electrophoresis on a 1.0% agarose gel with a NanoDrop®ND-2000 spectrophotometer (Thermo Scientific Inc., USA) and stored at − 80 °C until further use. The hypervariable region V3–V4 of the bacterial 16S rRNA gene was amplified using an ABI GeneAmp®9700 PCR thermocycler (ABI, CA, USA) (primer: 338F (5′-ACTCCTACGGGAGGCAGCAG-3′) and 806R (5′-GGACTACHVGGGTWTCTAA T-3′)). The PCR reaction system consisted of 4 μL of 5 × FastPfu buffer, 2 μL of 2.5 mM dNTPs, 0.8 μL of each primer (5 μM), 0.4 μL of FastPfu polymerase, 10 ng of template DNA, 0.2 μL of BSA, and ddH2O supplemented to a total volume of 20 μL. PCR amplification conditions were as follows: predenaturation at 95 °C for 3 min, followed by 27 cycles of denaturation at 95 °C for 30 s, annealing at 55 °C for 30 s, and extension at 72 °C for 45 s, followed by a single extension at 72 °C for 10 min and ending at 4 °C. All specimens were amplified in triplicate. PCR products were extracted in 2% agarose gels and purified using the AxyPrep DNA Gel Extraction kit (AxyPrep Biosciences, Union City, CA, USA) according to the instructions. The absolute 16S gene content in the specimens was determined by real-time PCR, using a ABI7300 Fluorescence Quantitative PCR Instrument (Applied Biosystems, USA). The PCR reaction system consisted of 10 μL of 2X Taq Plus Master Mix, 0.8 μL of each primer (5 μM), 1 μL of DNA template, and ddH2O supplemented to a total volume of 20 μL. PCR amplification conditions were as follows: predenaturation at 95 °C for 5 min, followed by 35 cycles of denaturation at 95 °C for 30 s, annealing at 58 °C for 30 s, and extension at 72 °C for 1 min and ending at 4 °C.

### Illumina MiSeq sequencing

Purified amplicons were pooled in equimolar amounts and paired-end sequenced on an Illumina MiSeq PE300 platform/NovaSeq PE250 platform (Illumina, San Diego, USA) according to the standard protocols by Majorbio Bio-Pharm Technology Co. Ltd. (Shanghai, China). The raw sequencing reads were deposited into the NCBI Sequence Read Archive (SRA) database (accession number: SRP435820).

### Data processing

Raw FASTQ files were multiplexed using in-house perl scripts, and then data were filtered using fastp version 0.19.6 and merged using FLASH version 1.2.7. with the following criteria:

(i) The 300-bp reads were truncated at any site receiving an average quality score of < 20 over a 50-bp sliding window, the truncated reads shorter than 50 bp were discarded, and reads containing ambiguous characters were also discarded.
(ii) Only overlapping sequences longer than 10 bp were assembled according to their overlapped sequence. The maximum mismatch ratio of overlap region is 0.2. Reads that could not be assembled were discarded.
(iii) Specimens were distinguished according to the barcode and primers, and the sequence direction was adjusted, exact barcode matching, and two nucleotide mismatches in primer matching. Then the optimized sequences were clustered into operational taxonomic units (OTUs) using UPARSE 7.1 [4, 5] with 97% sequence similarity level. The most abundant sequence for each OTU was selected as a representative sequence. To minimize the effects of sequencing depth on alpha- and beta-diversity measure, the number of 16S rRNA gene sequences from each specimens was rarefied to 20,000, which still yielded an average of good’s coverage of 99.09%, respectively.

### Statistical analysis

The specimen’s microbiome was subjected to bioinformatics analysis using Majorbio Cloud platform (https://cloud.majorbio.com). In order to obtain the species classification information corresponding to each OTUs, the Bayesian algorithm RDP classifier was used to perform taxonomic analysis on the 97% similarity level OTUs representative sequences, and the community species composition of each specimen was counted at each taxonomic level. Based on OTUs information, α diversity indexes including Sobs, Chao, Ace, Shannon, Simpson, and coverage were calculated using Mothur v1.30.1. Venn diagram was used to analyze the number of common and unique species in multiple groups or specimens to show the similarity and overlap of species composition in different specimens. Principal coordinate analysis (PCoA) based on Bray–Curtis distance algorithm in package Vegan v2.5–3 was used to determine the similarity of microbial communities in different specimens. Statistical significance was assessed by PLS-DA (partial least squares-discriminant analysis), using Vegan v2.5–3 package. The Kruskal–Wallis *H*-test was used to test the species between different groups of microbial communities to assess the significance level of differences in species abundance and to obtain significantly different species between groups. GraphPad Prism software was used to analyze and plot different data (*p* = 0.05 was used as the demarcation line for significance for all statistical tests).

## Results

### Assessment of lung and gut microbiome in LPS-induced ALI mice model

**Fig. 1:**
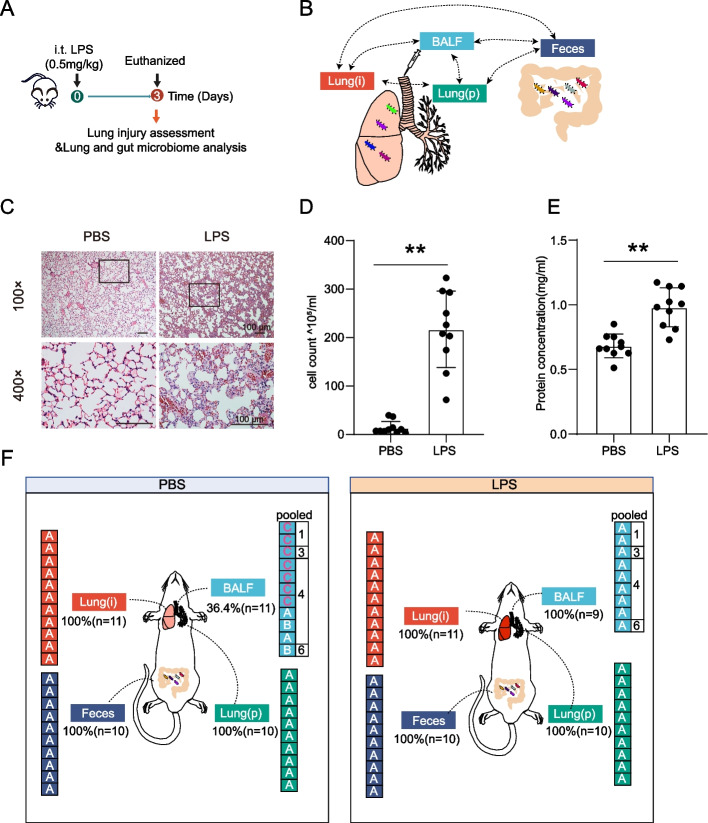
Assessment of lung and gut microbiome in LPS-induced ALI mice model. **A** Design of the animal LPS experiment. The mice were intratracheally administrated with LPS (lipopolysaccharide, 0.5 mg/kg), and then the microbial community compositions in lung and gut were determined by 16S rRNA gene sequencing 3 days later. **B** Schematic representation of specimen’s collection for testing. Bronchoalveolar lavage fluid (BALF), intact lung tissue [lung(i)], and lung tissue after perfused [lung(p)] were collected to assess lung microbiome, and feces were collected for the assessment of gut microbiome. The potential connections among these specimens were determined. **C** Representative H&E staining of lung tissues in the mice treated with LPS or PBS. Scale bar, 100 μm. **D** and **E** The BALF were obtained to determine the **D** cell count and **E** protein concentration (*n* = 10 for each group). **Indicated *P* < 0.01. **F** The DNA extraction quality of the lung and gut specimens. **A** and **B** represent the correct size of the target band of the PCR product, and the concentration is appropriate or slightly lower, which can be used for subsequent experiments. **C** represents the target band of the PCR product that was too weak or not detected for subsequent experiments (*n* = 9–11 mice in indicated group)

This study adopted a classical ALI mice model by intratracheal instillation of LPS (0.5 mg/kg) [25], and the control group was instilled with the same amount of PBS (Fig. 1A). To assess the compositions of lung microbiome, the lung microbiome was collected from three types specimens, including the intact lung tissues (did not perfused, hereafter referred as Lung(i)), bronchoalveolar lavage fluid (BALF), and the lung tissues after lavage (hereafter referred as Lung(p)). In addition, to determine whether gut-lung axis was involved in LPS-induced ALI, feces were collected for correlation analysis (Fig. 1B). The results of H&E staining of lung pathological slides showed that, compared with control group, increased inflammatory cells and thickened alveolar wall were detected in LPS treatment group (Fig. 1C), along with significant increases in cell counts and protein concentrations in BALF (Fig. 1D–E), together indicating the success of establishment of ALI mice model.

As expected, the microbiome DNA extracted from Lung(i) (*n* = 11), Lung(p) (*n* = 10), and feces (*n* = 10) were qualified, and the success ratios were 100% both in PBS and LPS groups (Fig. 1F). Intriguingly, the quality of microbiome DNA extracted from BALF was different in PBS and LPS groups. We noticed that all the microbiome DNA extracted from BALF of LPS groups were qualified, and the success ratios were 100%. However, in control groups, we failed to obtain qualified microbiome DNA from either individual mice BALF or three mice pooled BALF. The qualified microbiome DNA only can be extracted from four or six pooled mice BALF in control groups (Fig. 1F). We inferred that the low biomass of lung microbiome in control groups was responsible for the above results and which was seriously discussed below.

### Higher bacterial burden was observed in the lung specimens of LPS-induced ALI mice model

**Fig. 2:**
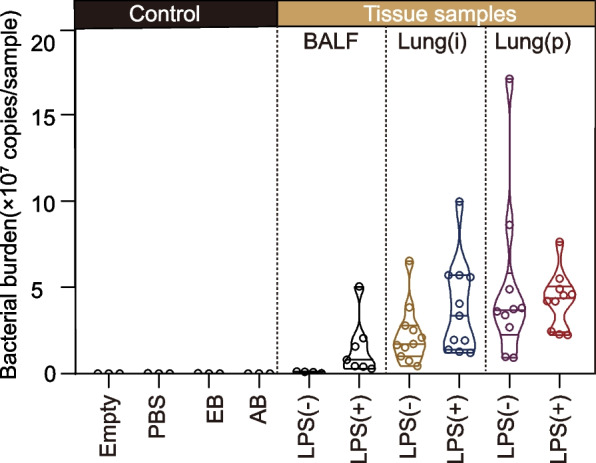
Bacterial DNA burden in the lung specimens obtained from LPS-induced ALI mice. Bacterial DNA burden in the lung specimens and controls were determined by qPCR method [*n* = 3 in control groups, *n* = 4 or 7 in BALF specimens, *n* = 11 in Lung(i) and *n* = 10 in Lung(P)]. Empty, empty EP tubes; PBS, the lavage solution; EB, DNA extraction buffer; AB, amplification buffer

To determine the presence and the burden of microbiome in the murine lung with or without LPS treatment, we extracted bacterial DNA from Lung(i), Lung(p), and BALF specimens. We performed real-time PCR on V3–V4 to obtain the 16S rRNA gene copy number for each specimen, by which the specimen’s bacterial load could be calculated. To exclude the possibility of DNA contaminations during the experimental process, we set empty EP tubes (Empty), PBS, DNA extraction buffer (EB), and amplification buffer (AB) as negative controls. As expected, the bacterial DNA in all the negative controls were negligible. In consistent with previous report [24], we also found that the bacterial DNA was higher both in Lung(i) and Lung(p) specimens than in the BALF specimens. Intriguingly, we noticed that the bacterial DNA in all the lung specimens with LPS treatment were elevated, suggesting that LPS treatment was efficient to increase the bacterial DNA in the lung specimens (Fig. 2).

### BALF, Lung(i), and Lung(p) specimens have similar alpha diversity of lung microbiome

**Fig. 3:**
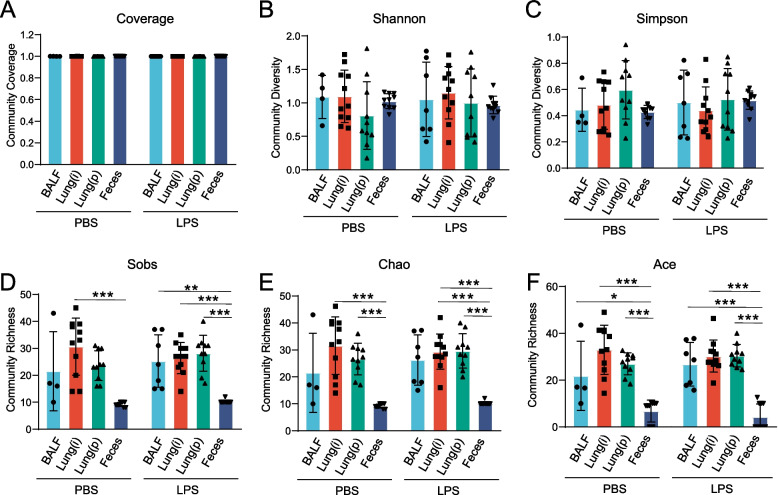
Alpha diversity of lung and gut microbiome in LPS-induced ALI mice. Alpha diversity of bacterial communities in the lung and gut specimens of mice treated with LPS or PBS. **A** Community coverage, **B** Shannon, **C** Simpson, **D** Sobs, **E** Chao, and **F** Ace indexes were presented [*n* = 4 or 7 in BALF specimens, *n* = 11 in Lung(i), *n* = 10 in Lung(P), and *n* = 10 in feces]. *Indicated *P* < 0.05, **indicated *P* < 0.01, and ***indicated *P* < 0.001

Alpha diversity analysis mainly evaluates information such as the richness and diversity of microbial communities through multiple diversity indices [28]. The microbial community profiles were generated according to clustering of the 16S rRNA sequences into OTUs (> 97% sequence match). The completeness of sequencing was assessed by coverage. The coverage indices were greater than 99%, indicating that most of the bacterial species were detected in the BALF, Lung(i), Lung(p), and feces specimens (Fig. 3A). The Shannon and Simpson indices indicated that similar community diversity among the BALF, Lung(i), Lung(p), and feces specimens (Fig. 3B and C). Sobs, Chao, and Ace indices indicated that similar community richness among the BALF, Lung(i), and Lung(p) specimens but lower community richness in feces specimens (Fig. 3D–F).

### Intact lung tissues and BALF had similar within-group variability and beta diversity

**Fig. 4:**
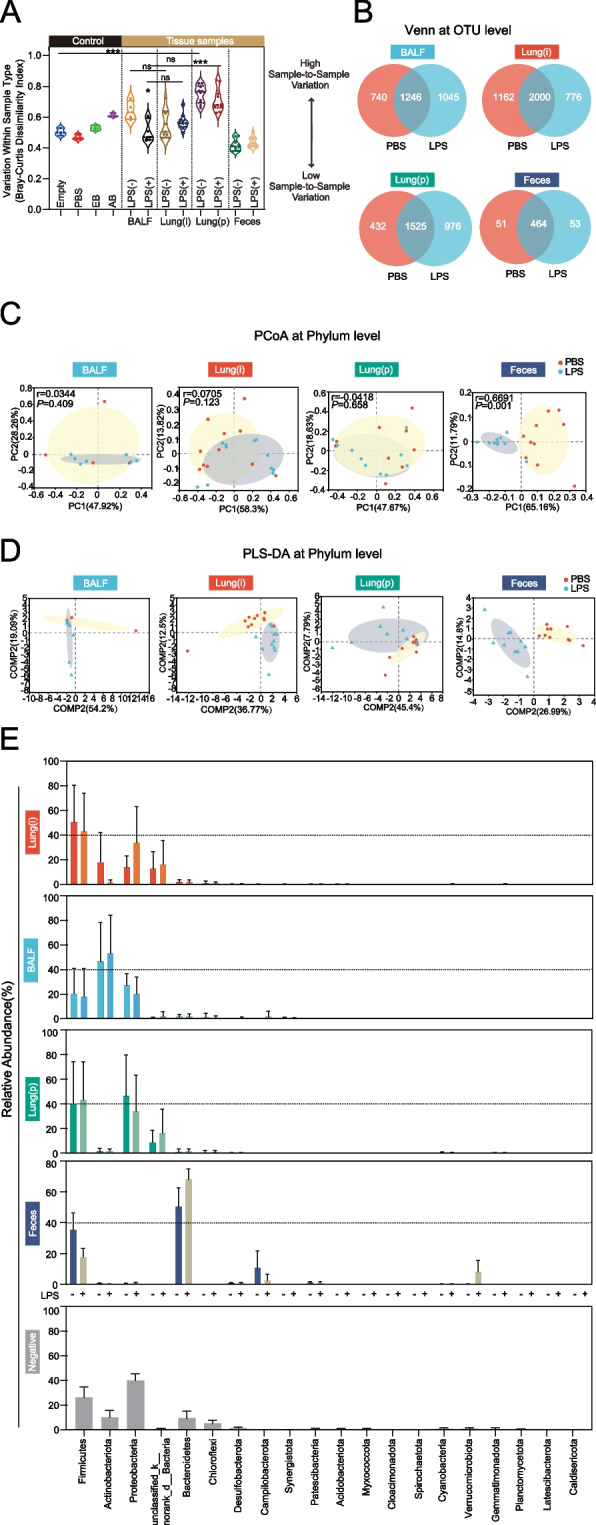
Beta diversity and species composition of lung and gut specimens in LPS-induced ALI mice. **A** Bray–Curtis dissimilarity index was calculated to reflect the replicability of lung and gut specimens. ns indicated not significant, *indicated *P* < 0.05, and ***indicated *P* < 0.001. **B** Venn diagram analysis at the OTU level. **C** PCoA and **D** PLS-DA at phylum level. **E** Results of the top 20 relative abundant phyla [ranked by Lung(i)] in lung and gut specimens. The phyla in negative were generated from control groups, including empty EP tubes, the lavage solution PBS, DNA extraction buffer, and amplification buffer [*n* = 3 in control groups, *n* = 4 or 7 in BALF specimens, *n* = 11 in Lung(i), *n* = 10 in Lung(P), and *n* = 10 in feces]

Bray–Curtis dissimilarity index, a beta-diversity metric based on pairwise inter-specimen distances between specimens of the same type, was applied to access the replicability of the lung specimens. In general, we found that the average Bray–Curtis dissimilarity index for feces specimen was lower than that of all lung specimens. Inconsistent with previous report, we did not observe any significant differences between BALF and Lung(i) specimens either in PBS or LPS treatment circumstances (BALF-PBS:0.64; BALF-LPS:0.51; Lung(i)-PBS:0.55; Lung(i)-LPS:0.57), suggesting that similar within-group variability between of BALF and Lung(i) specimens. Notably, the average Bray–Curtis dissimilarity index for Lung(p) specimen was higher than that of BALF and Lung(i) specimens (Lung(p)-PBS:0.75; Lung(p)-LPS:0.71), indicating that Lung(p) specimen had lower replicability (Fig. 4A).

The results of the Venn plot showed that BALF, Lung(i), and Lung(p) specimens contained similar OTUs (BALF-PBS:1986 OTUs, BALF-LPS:2291 OTUs, Lung(i)-PBS:3162 OTUs, Lung(i)-LPS:2776 OTUs, Lung(p)-PBS:1957 OTUs, Lung(p)-LPS:2501 OTUs), whereas the feces specimens contained the least OTUs (feces-PBS:515 OTUs; feces-LPS:517 OTUs). Venn diagram showed that different treatments (PBS or LPS) had an effect on the species composition of the specimens, which intuitively reflected the common and unique species in different groups. LPS treatment significantly changed the microbiome of all the lung specimens at the OTU level compared with PBS treatment, although the changes among the lung specimens were unapparent (BALF:1045OTUs; Lung(i):776OTUs; Lung(p):976OTUs) (Fig. 4B).

In order to visually observe the effects of different treatments on microbiome composition, we analyzed the beta diversity of specimens. PCoA analysis, or principal coordinate analysis, is a non-constrained data dimensionality reduction analysis method used to investigate the similarity or difference in community composition. The results of PCoA showed that LPS treatment had an effect on the lung microbiome community composition of all the lung specimens at the phylum level, although this effect did not reach the statistical significance (*P*-value: *BALF* = 0.409; Lung(i) = 0.123; Lung(p) = 0.658). Interestingly, the feces specimen’s microbiome community composition was significantly altered after LPS treatment (*r* = 0.6691, *P* = 0.001) (Fig. 4C). Partial least squares-discriminant analysis (PLS-DA) is a multivariate statistical analysis method used for discriminant analysis, which determines how to classify the research object according to the observed or measured values of several variables. Consistently, PLS-DA analysis got the similar results of PCoA analysis (Fig. 4D).

Since the obtain of lung microbiome from Lung(i) specimens was stable and reliable, rank abundance analysis was conducted according to the relative abundances in the Lung(i) specimens at phylum level. Remarkably, the mostly common microbiome was similar between Lung(i) and BALF specimens, including Firmicutes, Actinobacteriota, and Proteobacteria. However, the microbiome abundances in feces specimens were distinct from that in lung specimens, and the top ranked microbiome was Firmicutes, Bacteroidetes, and Campylobacterota (Fig. 4E).

### The change of Actinobacteriota was most significant in the microbiome of lung specimens

**Fig. 5:**
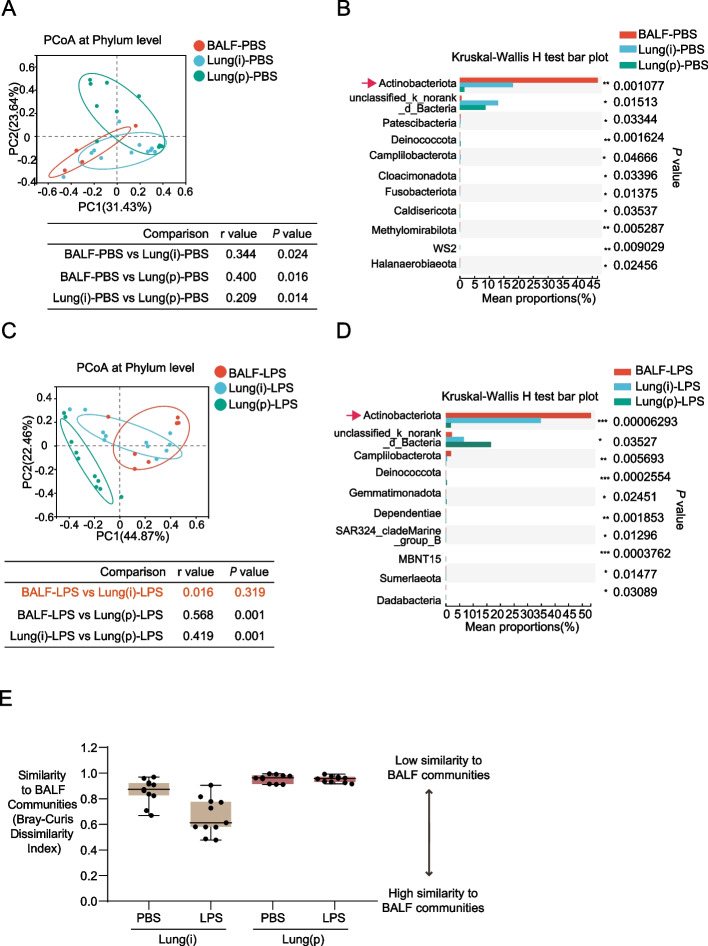
The results of PCoA and Kruskal–Wallis *H*-test bar plot in lung specimens. **A** PCoA and **B** Kruskal–Wallis *H*-test bar plot at phylum level in the lung specimens from mice treated with PBS [*n* = 4 in BALF specimens, *n* = 11 in lung(i), and *n* = 10 in lung(P)]. **C** PCoA and **D** Kruskal–Wallis *H*-test bar plot at phylum level in the lung specimens from mice treated with LPS [*n* = 7 in BALF specimens, *n* = 11 in Lung(i) and *n* = 10 in Lung(P)]. *Indicated *P* < 0.05, **indicated *P* < 0.01, and ***indicated *P* < 0.001. **E** Bray–Curtis dissimilarity index was calculated to reflect the replicability of Lung(i) and Lung(p) specimens compared with BALF specimens [*n* = 11 in Lung(i) and *n* = 10 in Lung(P)]

We further determined the differences of beta diversity among the lung specimens. The results of PCoA analysis showed that statistical significances existed among BALF, Lung(i), and Lung(p) specimens when treated with PBS (*P*-value: BALF-PBS vs Lung(i)-PBS = 0.024; BALF-PBS vs Lung(p)-PBS = 0.016; Lung(i)-PBS vs Lung(p)-PBS = 0.014) (Fig. 5A). Intriguingly, although the differences were still existed between BALF and Lung(p) (*P*-value: BALF-LPS vs Lung(p)-LPS = 0.001), Lung(i), and Lung(p) (*P*-value: Lung(i)-LPS vs Lung(p)-LPS = 0.001), the difference between BALF and Lung(i) specimens was disappeared when treated with LPS (*P*-value: BALF-LPS vs Lung(i)-LPS = 0.319) (Fig. 5C), suggesting that BALF-LPS and Lung(i)-LPS specimens have higher similarity. More importantly, the results of species difference analysis showed that among the lung specimens, the most changed microbiome at phylum level was Actinobacteriota in both the PBS (*P* = 0.001) and LPS groups (*P* < 0.0001) (Fig. 5B and D).

Due to BALF specimens are more convenient specimens obtained from patients compared with lung tissues, therefore, we determined the similarity of BALF specimens and lung tissue specimens. The results of Bray–Curtis dissimilarity index analysis showed that relative lower average Bray–Curtis dissimilarity index between Lung(i) specimens and BALF specimens, whereas relative higher average Bray–Curtis dissimilarity index between Lung(p) specimens and BALF specimens, suggesting BALF specimens and Lung(i) specimens are more suitable for the evaluation of lung microbiome than Lung(p) specimens (Fig. 5E).

### Potential connection between lung microbiome and gut microbiome

**Fig. 6:**
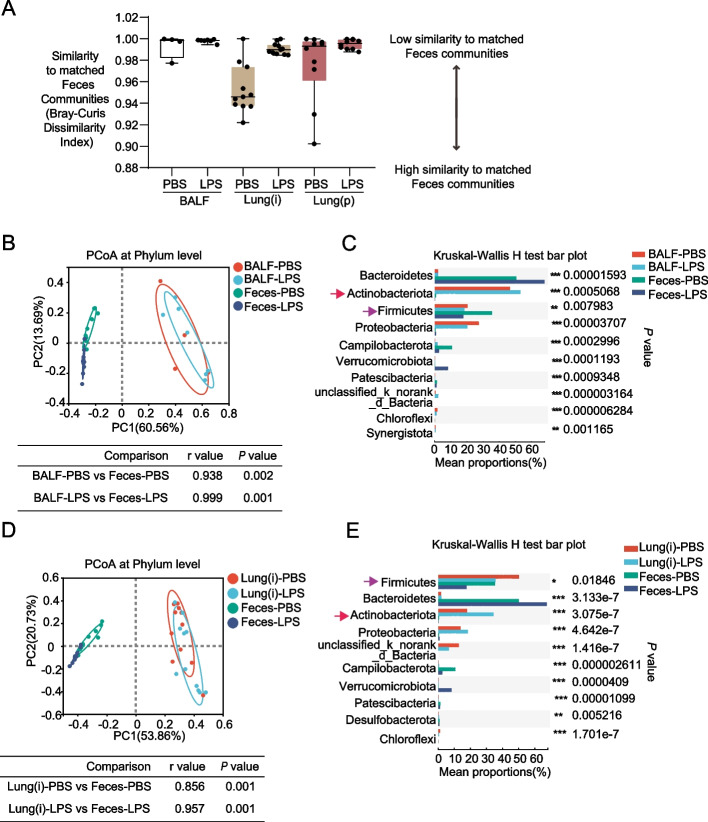
The results of PCoA and Kruskal–Wallis *H*-test bar plot in lung and gut specimens. **A** Bray–Curtis dissimilarity index was calculated to reflect the replicability of lung specimens compared with gut specimens [*n* = 4 or 7 in BALF specimens, *n* = 11 in Lung(i), and *n* = 10 in Lung(P)]. **B** PCoA and **C** Kruskal–Wallis *H*-test bar plot at phylum level in BALF and feces specimens from mice treated with PBS or LPS. **D** PCoA and **E** Kruskal–Wallis *H*-test bar plot at phylum level in Lung(i) specimens and feces from mice treated with PBS or LPS [*n* = 4 or 7 in BALF specimens, *n* = 11 in Lung(i), *n* = 10 in Lung(P), and *n* = 10 in feces]. *Indicated *P* < 0.05, **indicated *P* < 0.01, and ***indicated *P* < 0.001

The bidirectional communication hub between the gut and lung is called the gut-lung axis, which can affect the immune status of both organs. One of the well-accepted explanations is the interaction between lung microbiome and gut microbiome [29]. Therefore, we determined whether there exists the potential connection between lung microbiome (BALF and Lung(i) specimens) and gut microbiome (feces specimens). Firstly, Bray-Curis dissimilarity index was used to compare the dissimilarity between the lung specimens and the feces specimens (Fig. 6A). The results showed relative higher dissimilarity of either BALF or Lung(i) specimens with feces specimens, especially after the treatment of LPS (Fig. 6A). Although PCoA analysis showed that minimal overlap between the lung microbiome, and gut microbiome (Fig. 6B and D), in the Kruskal–Wallis *H*-test bar plot analysis, we can found that Firmicutes was reduced after LPS treatment in BALF, Lung(i), and feces specimens (*P* < 0.001 and* P* < 0.05) (Fig. 6C and E). Actinobacteriota was increased both in BALF and Lung(i) specimens, while decreased in feces specimens (*P* < 0.001) after LPS treatment (Fig. 6C and E), suggesting the potential connection between lung microbiome and gut microbiome, in particular Firmicutes and Actinobacteriota.

### Actinobacteriota and Proteobacteria might be correlated with the severity of lung injury

**Fig. 7:**
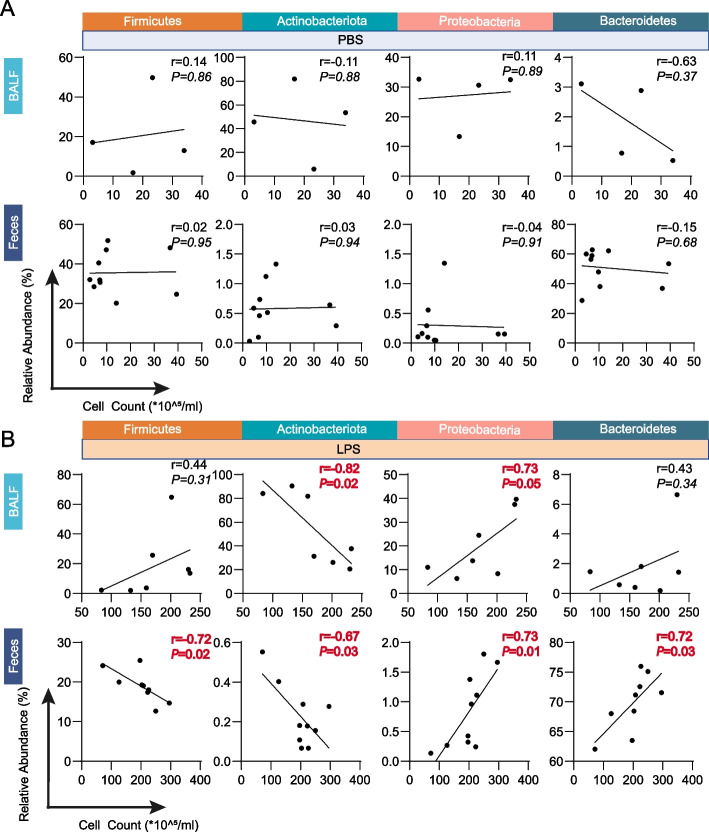
Correlation between the compositions of microbiome in BALF and feces specimens and severity of lung injury. The correlation between the relative abundance of microbiome at phylum level of BALF specimens and cell count in the mice treated with **A** PBS or **B** LPS [*n* = 4 or 7 in BALF specimens and *n* = 10 in feces]

We further explore whether the lung or/and gut microbiome can be used to predict the severity of lung injury. We chose Actinobacteriota, Proteobacteria, Firmicutes, and Bacteroidetes, the most changed microbiome at phylum levels according to the results shown in Fig. 6B and D, to correlate with the cell count index in BALF. Due to the cell counts are not obtained in Lung(i) specimens, therefore, only BALF and feces specimens are applied for this assessment. The results showed that in the PBS-treated group, there was little correlation between microbiome and cell counts, either in BALF or feces specimens (Fig. 7A). Interestingly, the correlation between gut and lung microbiome and cell counts was enhanced in the LPS-treated group. Firmicutes, Proteobacteria, and Bacteroidetes were positively correlated with cell counts in BALF, with correlation coefficients of 0.44, 0.73, and 0.43, respectively. Actinobacteriota was negatively correlated with cell counts, with a correlation coefficient of − 0.82. In feces specimens, Proteobacteria and Bacteroidetes were positively correlated with cell count, with correlation coefficients of 0.73 and 0.72, respectively. Firmicutes and Actinobacteriota were negatively correlated with cell count, with correlation coefficients of − 0.72 and − 0.67, respectively. These findings suggest that there is a potential correlation between the microbiome (particularly *Actinobacteriota* and *Proteobacteria*) and the severity of lung injury. However, the underlying mechanisms need to be further explored.

## Discussion

The key findings in the current study include the following: (1) we demonstrated that BALF and Lung(i) specimens were both suitable, but Lung(p) was not suitable, for the assessment of lung microbiome in ALI mice model, (2) Actinobacteriota was the most changed bacterial phylum in the lung, (3) Actinobacteriota and Firmicutes might be the important factors involved in the gut-lung axis in ALI mice model, (4) Actinobacteriota and Proteobacteria might serve as potential biomarkers for the severity of lung injury.

The biomass of the lung microbiome is very low compared to the gut microbiome [30]. Our study confirms the presence of a lung microbiome that is increased in LPS-induced ALI. Under normal circumstances, most of the microbiome in the lung originates from inhalation or micro-aspiration from the upper respiratory tract, by which the total biomass inhaled and exhaled, and the relative reproduction of microorganisms maintains a dynamic balance [30]. The presence of microbiome in healthy lungs is transient and changeable compared with gut microbiome, whereas when disease occurs, it leads to the dysbiosis of lung microbiome and the colonization of pathogenic bacteria [5, 31]. Dickson et al., in their study of the lung microbiome of healthy mice, showed that the lung microbiome of mice varied according to the manufacturer and the lot purchased [23]. In our study, we adopted a strategy that is pooling BALF specimens from multiple mice to address the issue of the low biomass of the lung microbiome. We found that when pooling BALF specimens from four or six PBS-treated mice, the successful detection rate began to increase. In other words, BALF specimens are also suitable for the evaluation of lung microbiome when enough BALF was obtained from untreated mice. It is worth mentioning that LPS treatment can significantly increase lung microbiome, because the BALF obtained from one LPS-treated mouse was enough for 16S rRNA detection. As such, in the current study, we provide the insight that the BALF specimens are suitable for the detection of lung microbiome in ALI study or other lung disease researches (Table 1).

**Table 1:** Summary of lung specimens for the evaluation of lung microbiome

		BALF		Lung(i)		Lung(p)		Feces	
		PBS	LPS	PBS	LPS	PBS	LPS	PBS	LPS
Basic description	Clinical availability	Easy	Easy	Hard	Hard	Hard	Hard	Easy	Easy
Summary of findings (relative to each other)	Bacterial biomass	Low	Medium	Medium	Medium	Medium	Medium	High	High
	Total DNA quality	Dependent	Good	Good	Good	Good	Good	Good	Good
	Bacterial DNA content	Low	Medium	Medium	High	High	High	——	——
	Results of alpha diversity	Medium	Medium	Medium	Medium	Medium	Medium	Low	Low
	Variation among biological replicates	Medium	Medium	Medium	Medium	High	High	Low	Low
	Similarity to biological source community (feces microbiome)	Low	Low	Low	Low	Low	Low	——	——
	Potential ALI biomarker	No	Potential	——	——	——	——	No	Potential

Notably, intact lung tissues and BALF both have the advantages and disadvantages to evaluate lung microbiome. On one hand, intact lung tissue analysis allows for a more comprehensive assessment of the lung microbiome by capturing the microbial communities residing within the tissue. However, it requires invasive sampling and may not reflect the microbial composition in the airways directly. On the other hand, BAL fluid analysis provides a more localized assessment of the microbial communities in the airways, but it may not capture microbes residing deeper in the lung tissue. Sampling methods, processing, and analysis techniques may affect the results. BALF sampling methods should be standardized to ensure more accurate and repeatable results. For murine BALF samples, the lungs were perfused three times through the trachea with 1-mL PBS. During each perfusion, the lungs should be expanded with PBS for 30 s with gently compression, and the recovery rate of BLAF should exceed 60–80%. In the process of clinical surgery, the collection of BALF can be affected by the state of the patient, the technical level of the surgical operator, the time and method of collection, etc. In order to make full utilize of the detection value of BALF, the accepted operation for standard BALF sampling process includes the following: 1 ~ 2 mL 2% lidocaine was injected into the perfused lung segment through the biopsy hole by a thin silicone tube for local anesthesia. Then, the fiber-optic bronchoscope was tightly wedged into the opening of the target bronchial segment or subsegment, and 37 °C sterilized saline was rapidly injected through the silicone tube by the biopsy hole, with a total volume that is between 100 and 240 mL using multiple aliquots of 20–60 mL (usually four to five aliquots). After instillation, the liquid is immediately aspirated and recovered, using a negative suction pressure less than 100 mmHg. Ideally, the percentage of recovered BAL fluid should be at least 30% of the instilled fluid [32, 33]. During this procedure, the tip of the bronchoscope should be tightly embedded in the opening of the segmental or subsegmental bronchus to prevent the infiltration of airway secretions or the spillage of lavage fluid. Nevertheless, the selection of intact lung tissue or BALF to assess lung microbiome may not be mutually exclusive, and the optimized strategy should be case dependent.

Previous studies have provided several explanations for why the biomass of lung microbiome increased in pathological conditions. In Dickson et al., they speculated that in a specific pathological state, the surface area of the lung was reduced, thus providing a growth environment and promoting the reproduction of pathogenic bacteria [3]. O'Dwyer et al. suggested that changes in local mucosal conditions in conjunction with comorbidities may alter the composition and burden of lung microbiota [34]. Wong-Rolle et al. pointed out that Wnt/β-catenin, hypoxia, and angiogenesis pathways showed a strong positive correlation with bacterial burden in lung tumor cells [35]. Invernizzi and colleagues reported an absence of correlations between key radiological markers and physiological features of IPF and lung bacterial burden, demonstrating that the increased bacterial burden reported in IPF was not simply the direct result of architectural distortion and parenchymal destruction [36]. In spite of the above studies providing clues for the explanation of the increased lung bacterial burden under lung disease conditions, the detailed mechanism still largely unknown. In addition, because Lung(p) specimen had lower replicability (as evidenced by Bray–Curtis dissimilarity index) and distinct beta diversity profile compared with BALF or Lung(i) specimens (as evidenced by PCoA analysis), together indicating that Lung(p) was not suitable for the detection of lung microbiome.

The interaction between the gut and lung is termed as the gut-lung axis [37]. At present, one of the well-accepted explanations of the gut-lung axis is that in diseases dominated by one organ of the intestine or lung, the other organ also produces corresponding symptoms. Limited evidence suggests that the translocation of gut bacteria to lung might be responsible for the gut-lung axis. Dickson et al. determined the gut and pulmonary microbiome of cecal ligation and puncture (CLP) mice and acute respiratory distress syndrome (ARDS) patients and found that specific gut bacterial (*Bacteroides*) were enriched in the lungs and closely associated with systemic inflammation [38]. In addition, Panzer et al. demonstrated that the development of ARDS was associated with the composition of lung communities enriched for Enterobacteriaceae (belong to Proteobacteria), which is both commensal and pathogenic in the human gut [39]. Narayana et al. pointed out that microbiome (such as pulmonary *Pseudomonas*, intestinal *Bacteroides*, and intestinal yeasts) in bronchiectasis exhibited significant gut-lung interactions and provided evidence that the presence of *Pseudomonas aeruginosa* in the airways could have an impact on the gut microbiome [40].

We now found that *Firmicutes* was reduced after LPS treatment in BALF, Lung(i), and feces specimens, whereas *Actinobacteriota* was increased both in BALF and Lung(i) specimens but decreased in feces specimens after LPS treatment, suggesting the potential connection between lung microbiome and gut microbiome. *Lactobacillus murinus* are one of the most important probiotics in the gut microbiome and belong to the Firmicutes phylum. We found that *Lactobacillus murinus* were reduced after LPS treatment in BALF, Lung(i), and feces specimens (Figure E2A and B). *Lactobacillus* or its components, such as peptidoglycan, metabolites, and surface proteins, have been shown to play immunomodulatory roles in the treatment of patients with chronic respiratory diseases [41]. Importantly, in animal studies and clinical trials, administration of specific Lactobacilli could alleviate symptoms of respiratory diseases, such as respiratory infections, asthma, lung cancer, and cystic fibrosis [42]. Why intratracheal instillation of LPS resulted in the alteration of gut microbiome? Several explanations should be accounted for this issue. Firstly, LPS intratracheal instillation causes lung injury and inflammation, resulting in increased permeability and inflammatory factors, which can enter into the circulation and then reach to gut to affect the intestinal microbiota. Secondly, the lung injury and inflammation were caused by LPS intratracheal instillation, which directly leads to the changes of lung microbiota. Recently, Leon and colleagues reported that the lung microbiome regulated brain autoimmunity, because lung dysbiosis affected microglia immune reactivity, therefore presenting a novel insight that distal effects of lung microbiome on other organs [43]. According to this finding, it is assumed that lung microbiome alteration is able to influence gut microbiome, although the mechanism is still obscure. Thirdly, in turn, it is also possible that changes in gut microbiota under inflammatory conditions would influence lung microbiome, through translocation of specific bacteria or secreting gut microbiota-derived metabolism to lungs [38, 39, 44]. Indeed, in the previous study from our team, we demonstrated that intestinal microbiota-derived propionic acid protects against zinc oxide nanoparticles pulmonary exposure-induced acute lung injury [45].

Intriguingly, besides Firmicutes, Actinobacteriota was involved in the gut-lung axis and also the most changed bacterial phylum in the lung microbiome. *Rhodococcus erythropolis*, which belonged to Actinobacteria phylum, had the most significant change both in the lung microbiome of mice treated with PBS or LPS at species level (Figure E1A and B). Carvalho and da Fonseca pointed out that *Rhodococcus erythropolis* contained a large number of enzymes that play important roles in oxidation, dehydrogenation, and desulfurization, manifesting the potential applications of this bacterium in biomedical fields [46]. Osoagbaka provided evidence of the pathogenic role of *Rhodococcus* in pulmonary disease by culturing strains isolated from sputum of patients with chest disease [47]. *Rhodococcus* infections occur predominantly in immunocompromised patients, and multiple infectious complications have been noted in patients treated with rituximab and methotrexate [48]. The role and underlying mechanisms that Firmicutes and Actinobacteriota participated in the process of lung injury should be further investigated.

In our study, Firmicutes, Actinobacteriota, Proteobacteria, and Bacteroidetes were found to be associated with the severity of lung injury, in particular Actinobacteriota and Proteobacteria. A significant reduction in the relative abundance of Firmicutes and Actinobacteria in lungs were found in children at risk of asthma [49]. Huang et al. pointed out that significant enrichment of Actinobacteria at phylum level and *Klebsiella* (which belongs to Proteobacteria) at genus level, in patients with severe asthma compared with healthy controls or patients with asthma [12]. *Pseudomonas aeruginosa* (which belongs to Proteobacteria) is an important opportunistic pathogen which might cause ALI/ARDS; it was the predominant species isolated from patients with nosocomial infections and was detected in almost all patients with prolonged ventilation in the intensive care unit [50]. In the COPD mouse model constructed by Yadava et al., the relative abundance of *Pseudomonas aeruginosa* increased in the BALF of LPS/elastase-treated mice and was associated with the severity of COPD [27]. Strikingly, intervention of the lung microbiota with antibiotics significantly affected the susceptibility of rats to autoimmune diseases of the central nervous system, suggesting that lung microbiome regulates distal organ diseases and therefore serves as potential intervention target [43]. However, the notion is that manipulating lung microbiome for the therapy of lung injury needs to be validated in future [51, 52].

Several limitations exist in the current study. Firstly, murine models of acute lung injury may not perfectly replicate the complexity and diversity of human lung conditions. The murine lung microbiome might differ from the human lung microbiome in terms of microbial composition and functional potential. Additionally, the immune response and other physiological differences between mice and humans can influence the lung microbiome dynamics and its association with acute lung injury. Secondly, we pooled BALF specimens from multiple mice to address the issue of that low biomass of the lung microbiome. The lung microbiome of the control group was not identified until the mixture number reached to 4 or even 6, which led to inconveniences for experiments. Therefore, optimized BALF collection and handling methods or alternative animal model (such as rat or pig) might be more suitable for the assessment of lung microbiome in native circumstance. In fact, we almost successfully obtained enough lung microbiome for 16S rRNA sequencing from 15 patients without ARDS. It is mentioned that due to the relatively small number in BALF-PBS group (*n* = 4), it might induce statistical inefficiency. Thirdly, we performed data mining on the results primarily at the phylum level, which may lead to incomplete conclusion. Huang et al. pointed out significant enrichment of Actinobacteria in patients with severe asthma compared to healthy controls or patients with mild to moderate asthma, although the largest differences observed at genus level were *Klebsiella*, which belonged to Proteobacteria phylum [12]. Lastly, this study lacks the evidence from antibiotic intervention, fecal microbiota transplantation, or single bacterial strain transplantation, which should be conducted in our following studies.

In summary, we present evidence to support the concept of that applicability of both BALF and Lung(i) specimens for determining murine lung microbiome in ALI. Several phyla (such as Actinobacteriota) may serve as potential biomarkers for the severity of lung injury.

### Supplementary Information

**Additional file 1:** **Figure E1.** Kruskal-Wallis H test bar plot at species level in lung specimens. Kruskal-Wallis H test bar plot at species level in lung specimens from the mice treated with (A) PBS or (B) LPS [*n*=4 or 7 in BALF specimens, *n*=11 in Lung(i) and *n*=10 in Lung(P)]. *indicated *P*<0.05, **indicated *P*<0.01, and ***indicated *P*<0.001. **Figure E2.** Kruskal-Wallis H test bar plot at species level in BALF, Lung(i) and feces specimens. (A) Kruskal-Wallis H test bar plot at species level in BALF specimens and feces from mice treated with PBS or LPS [*n*=4 or 7 in BALF specimens and *n*=10 in Feces]. (B) Kruskal-Wallis H test bar plot at species level in Lung(i) specimens and feces from mice treated with PBS or LPS [*n*=11 in Lung(i) specimens and *n*=10 in Feces]. *indicated *P*<0.05, **indicated *P*<0.01, and ***indicated *P*<0.001.

**Additional file 2:** **Table E1.** Overall sample delivery situation. **Table E2.** Sample delivery and success rate of BALF,Lung (i) and Lung (p). **Table E3.** Sample delivery and success rate of Feces. **Table E4.** Sample delivery and success rate of negative controls.

## Data Availability

Data and materials may be made available upon written request to the corresponding author.

## Abbreviations

ALI: Acute lung injury
ARDS: Acute respiratory distress syndrome
BALF: Bronchoalveolar lavage fluid
COPD: Chronic obstructive pulmonary disease
H&E staining: Hematoxylin-eosin staining
IPF: Idiopathic pulmonary fibrosis
LPS: Lipopolysaccharide
Lung(i): Intact lung tissues
Lung(p): Perfused lung tissue
OTUs: Operational taxonomic units
PBS: Phosphate buffer saline
PCoA: Principal coordinates analysis
PLS-DA: Partial least squares discriminant analysis
16S rRNA: 16S ribosomal RNA
