# Supplementary figures and images for: Intact lung tissue and bronchoalveolar lavage fluid are both suitable for the evaluation of murine lung microbiome in acute lung injury
Source: Microbiome. 2024 Mar 18;12:56. doi: 10.1186/s40168-024-01772-6 (PMC10946114; doi:10.1186/s40168-024-01772-6)

Figure E1

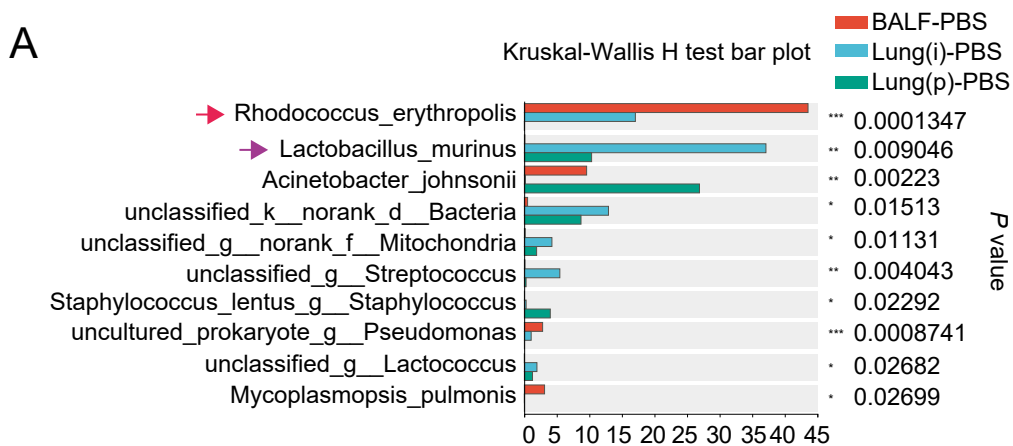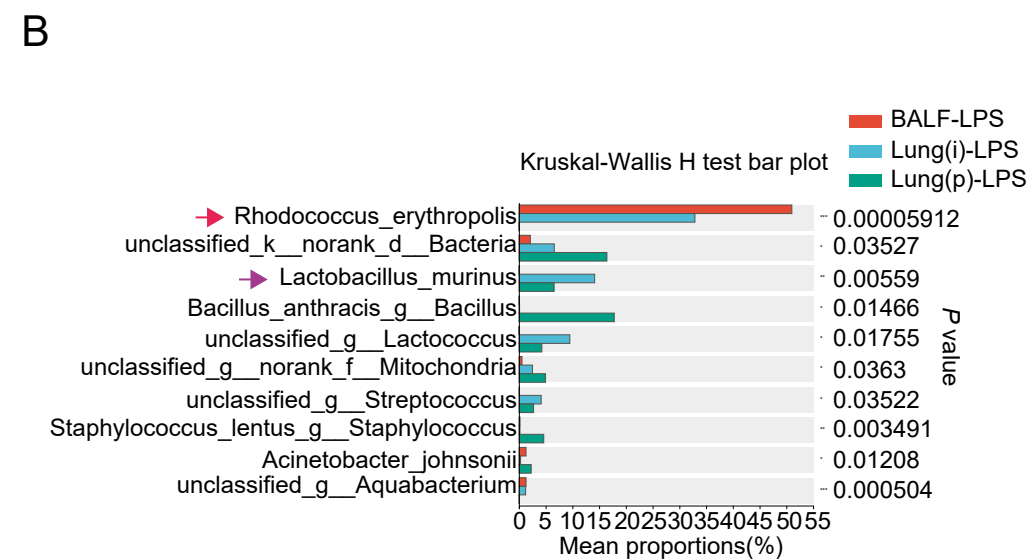

Figure E2

A

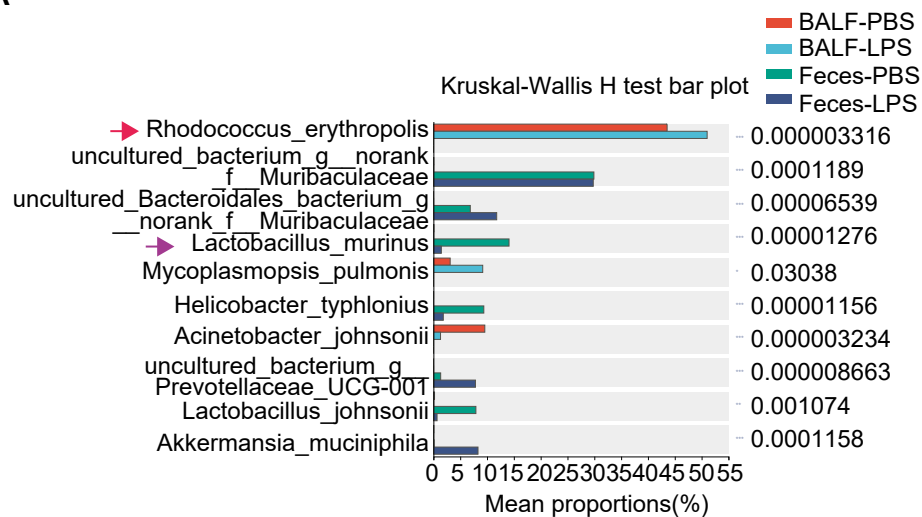

B

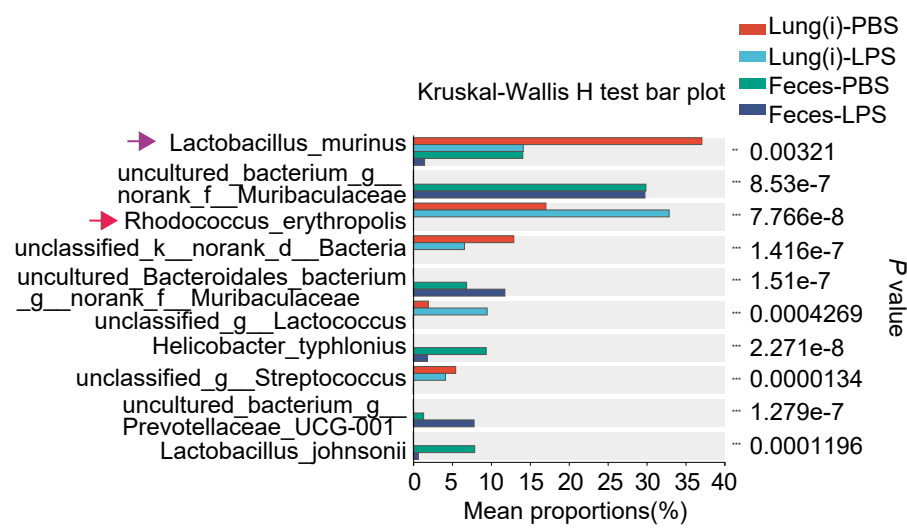

Supplement: Supplementary file 2 — Additional file 1: Figure E1. Kruskal-Wallis H test bar plot at species level in lung specimens. Kruskal-Wallis H test bar plot at species level in lung specimens from the mice treated with (A) PBS or (B) LPS [n=4 or 7 in BALF specimens, n=11 in Lung(i) and n=10 in Lung(P)]. *indicated P<0.05, **indicated P<0.01, and ***indicated P<0.001. Figure E2. Kruskal-Wallis H test bar plot at species level in BALF, Lung(i) and feces specimens. (A) Kruskal-Wallis H test bar plot at species level in BALF specimens and feces from mice treated with PBS or LPS [n=4 or 7 in BALF specimens and n=10 in Feces]. (B) Kruskal-Wallis H test bar plot at species level in Lung(i) specimens and feces from mice treated with PBS or LPS [n=11 in Lung(i) specimens and n=10 in Feces]. *indicated P<0.05, **indicated P<0.01, and ***indicated P<0.001. [file 40168_2024_1772_MOESM1_ESM.pdf]
