# Supplementary material for: Intact lung tissue and bronchoalveolar lavage fluid are both suitable for the evaluation of murine lung microbiome in acute lung injury
Source: Microbiome. 2024 Mar 18;12:56. doi: 10.1186/s40168-024-01772-6 (PMC10946114; doi:10.1186/s40168-024-01772-6)
Supplement: Supplementary file 3 — Additional file 2: Table E1. Overall sample delivery situation. Table E2. Sample delivery and success rate of BALF,Lung (i) and Lung (p). Table E3. Sample delivery and success rate of Feces. Table E4. Sample delivery and success rate of negative controls. [file 40168_2024_1772_MOESM2_ESM.docx]

**Table E1:** **Overall sample delivery situation**

| Lung Microbiome | BALF | | Lung (i) | | Lung (p) | | Feces | | Total |
| --- | --- | --- | --- | --- | --- | --- | --- | --- | --- |
|  | PBS | LPS | PBS | LPS | PBS | LPS | PBS | LPS |  |
| experiment 1 | 0(2) | 2(2) | - | - | - | - | - | - | 2(4) |
| experiment 2 | 1(1) | 1(1) | - | - | - | - | - | - | 2(2) |
| experiment 3 | 2(5) | 3(3) | 1(1) | 1(1) | - | - | - | - | 7(10) |
| experiment 4 | 1(3) | 3(3) | 10(10) | 10(10) | 10(10) | 10(10) | 10(10) | 10(10) | 64(66) |
| Total | 4(11) | 9(9) | 11(11) | 11(11) | 10(10) | 10(10) | 10(10) | 10(10) | 75(82) |
| success rate | 0.36 | 1 | 1 | 1 | 1 | 1 | 1 | 1 | 0.91 |

**Table E2: Sample delivery and success rate of BALF,Lung (i) and Lung (p).**

| Lung Microbiome | BALF | | | |
| --- | --- | --- | --- | --- |
| treatment | PBS | success rate | LPS | success rate |
| n=1 | 0(2) | 0 | 2(2) | 1 |
| n=3 | 0(1) | 0 | 1(1) | 1 |
| n=4 | 3(7) | 0.43 | 5(5) | 1 |
| n=6 | 1(1) | 1 | 1(1) | 1 |
| Total | 4(11) | 0.36 | 9(9) | 1 |
|  | **Lung (i)** | | | |
| treatment | PBS | success rate | LPS | success rate |
| n=1 | 11(11) | 1 | 11(11) | 1 |
| Total | 11(11) | 1 | 11(11) | 1 |
|  | **Lung (p)** | | | |
| treatment | PBS | success rate | LPS | success rate |
| n=1 | 10(10) | 1 | 10(10) | 1 |
| Total | 10(10) | 1 | 10(10) | 1 |

**Table E3: Sample delivery and success rate of Feces.**

| Gut Microbiome | Feces | | | |
| --- | --- | --- | --- | --- |
|  | PBS | success rate | LPS | success rate |
| n=1 | 10(10) | 1 | 10(10) | 1 |
| Total | 10(10) | 1 | 10(10) | 1 |

**Table E4: Sample delivery and success rate of** **negative controls.**

| Sample name | sample types | Concentration (ng/μl) | OD 260/280 | OD 260/230 | result |
| --- | --- | --- | --- | --- | --- |
| Empty 1 | Air | 4.52 | 1.23 | 0.01 | C |
| Empty 2 | Air | 5.23 | 1.37 | 0.01 | C |
| Empty 3 | Air | 5.23 | 1.37 | 0.01 | C |
| PBS 1 | PBS | 3.08 | 1.35 | 0.01 | C |
| PBS 2 | PBS | 1.00 | 1.21 | 0.01 | C |
| PBS 3 | PBS | 1.24 | 1.26 | 0.01 | C |
| Extraction Buffer 1 | buffer | 1.16 | 1.61 | 0.01 | C |
| Extraction Buffer 2 | buffer | 2.29 | 1.65 | 0.01 | C |
| Extraction Buffer 3 | buffer | 3.09 | 1.64 | 0.01 | C |
| Amplification buffer1 | buffer | 1.14 | 1.93 | 0.01 | C |
| Amplification buffer2 | buffer | 6.04 | 1.65 | 0.01 | C |
| Amplification buffer3 | buffer | 1.28 | 1.62 | 0.02 | C |
